# Levels of biological plausibility

**DOI:** 10.1098/rstb.2019.0632

**Published:** 2020-11-16

**Authors:** Bradley C. Love

**Affiliations:** University College London, Gower Street, London WC1E 6BT, UK

**Keywords:** levels of analysis, biological plausibility, emergence, reduction, mechanism, model selection

## Abstract

Notions of mechanism, emergence, reduction and explanation are all tied to levels of analysis. I cover the relationship between lower and higher levels, suggest a level of mechanism approach for neuroscience in which the components of a mechanism can themselves be further decomposed and argue that scientists' goals are best realized by focusing on pragmatic concerns rather than on metaphysical claims about what is ‘real'. Inexplicably, neuroscientists are enchanted by both reduction and emergence. A fascination with reduction is misplaced given that theory is neither sufficiently developed nor formal to allow it, whereas metaphysical claims of emergence bring physicalism into question. Moreover, neuroscience's existence as a discipline is owed to higher-level concepts that prove useful in practice. Claims of biological plausibility are shown to be incoherent from a level of mechanism view and more generally are vacuous. Instead, the relevant findings to address should be specified so that model selection procedures can adjudicate between competing accounts. Model selection can help reduce theoretical confusions and direct empirical investigations. Although measures themselves, such as behaviour, blood-oxygen-level-dependent (BOLD) and single-unit recordings, are not levels of analysis, like levels, no measure is fundamental and understanding how measures relate can hasten scientific progress.

This article is part of the theme issue ‘Key relationships between non-invasive functional neuroimaging and the underlying neuronal activity'.

## 1. Introduction

Although levels of analysis are frequently discussed in neuroscience, cognitive science and philosophy, widespread confusion persists over what a level is and how various levels relate to one another [1,2]. This confusion is a headwind to scientific progress because it leads to misplaced claims about which data sources are fundamental and what is biologically plausible. Here, I consider what is gained and lost across various levels of analysis. A firm conceptual grasp of levels of analysis is necessary for common terms in neuroscience to have meaning. Notions of mechanism, biological plausibility, emergence and reduction are all tied to levels of analysis.

In this contribution, the connections between key concepts in neuroscience and levels of analysis will be unpacked. I will consider whence levels in neuroscience arise and whether neural measures at different granularities, such as cellular versus blood-oxygen-level-dependent (BOLD) response, constitute different levels of analyses. Other limits on the applicability of levels of analyses will be considered. In particular, I will suggest that claims of biological plausibility are better cast as (and resolved through) model selection than by appeal to the level of analysis that makes contact with ‘true' biology. Indeed, the latter position, while common in neuroscience, will be shown to be incoherent. Under the best of circumstances, claims of biological plausibility do not offer value beyond what could be gained from model selection procedures, which specify the relevant findings and competing accounts. In other cases, claims of biological plausibility can be vacuous and lead to confusion.

Marr's tripartite hierarchy [3] is perhaps the most well-known and influential organization of levels in neuroscience. In brief, the computational level is the top level where the problem to be addressed is specified. Rather than detail the form of a potential solution, the computational level simply states the problem (i.e. the input–output mapping desired). For example, for object recognition, a computational level account could involve naming various images under various conditions. The next level is the algorithmic level. As its name indicates, the algorithmic level is concerned with how the function specified at the computational level is computed (i.e. the processes and representations used). For example, if the computational level task were to sort an array of numbers in ascending order, then the algorithmic level would specify a possible approach, such as bubble sort or quicksort. Different algorithms may solve the computational task in different ways, have different runtimes, etc., but they should all conform to the computational level goal (e.g. correctly sort the array). Finally, the implementational level describes the physical substrate for the computation (e.g. the computer that executes quicksort).

**Figure 1.**
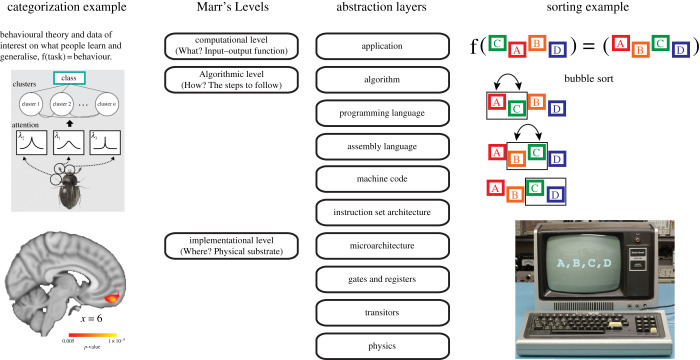
Marr's levels compared to abstraction layers in computing with examples of each. Marr's levels are clearly influenced by abstraction layers in computer science, though Marr's levels are less fine grain, particularly for levels of interest to many neuroscientists. On the left, an example from category learning is shown in which an algorithmic model [5] was fit to behaviour and its internal representations are used to interpret BOLD response [6]. On the right, a sorting algorithm addressed the computational level problem of sorting and was implemented by a digital computer. The abstraction layers in computing make clear that moving to a lower layer introduces additional detail (more information) about the computation whereas higher layers introduce abstract constructs that can be realized in multiple ways. (Online version in colour.)

The previous examples from computer science are apropos as Marr was clearly inspired by abstraction layers, a central concept in computer science [4]. Note that Marr's top two levels, the computational and algorithmic, neatly map onto the top two levels in a common abstraction hierarchy in computing (figure 1). Abstraction layers in computing can contain finer-grain levels, including multiple levels describing the physical computing device. By contrast, Marr effectively lumped all of neuroscience into a single implementational level, which might partly explain why some neuroscientists find his hierarchy inadequate [7].

Although Marr's scheme is highly influential, there are alternatives [8]. Moreover, there is no reason to restrict to three levels. For example, there are a number of four-level schemes in cognitive science [9–12]. Indeed, Bechtel & Richardon's [13] mechanistic approach can be characterized as a ‘levels of mechanism' hierarchy in which there is not a fixed number of levels. For example, a car can be seen as a mechanism consisting of interacting parts, such as an engine, drivetrain, steering wheel and brakes. What is a component of a mechanism itself can be further decomposed into its own mechanism (e.g. braking system) and so forth, with no limit except those imposed by particle physics.

The mechanistic decomposition approach was conceived with an eye toward explanation in biology. Craver [14] uses an example from neuroscience to motivate the levels of mechanism view, which contains multiple levels of mechanism: namely spatial memory, spatial map formation, cellular and molecular levels. Like the car example, a component in the mechanism at one level can itself decompose at the next lower level [15]. For example, at the molecular level, an NMDA receptor is a component of the LTP mechanism, which in turn is a component of the hippocampus, which in turn is a component in spatial memory.

Although theories in neuroscience can fall short (see [16] for criticisms of some prominent imaging work in cognitive neuroscience), most neuroscientists would seem to aspire to this type of multi-level mechanistic explanation. Notice that in such a multi-level explanation, there is no level that is more biologically plausible or preferred in some general sense. Although there can be a tendency in neuroscience to dismiss higher level explanations (e.g. a cognitive model) as biologically implausible or not real in some sense, this makes as much sense as stating that a sorting algorithm, a car's engine, the heart, or the hippocampus is not real because it can be further decomposed. In other fields, such as economics, the status of macroeconomics, which is concerned with aggregate activity in the economy, is not threatened by the existence of microeconomics, which is concerned with the behaviour of individuals and firms that give rise to the aggregate. Recently, there has been a call for neuroscientists to reconsider their reductionist biases in the interest of scientific progress [17].

Of course, building multi-level explanations is challenging. For example, mechanisms will rarely decompose neatly into localizable, independent components [13]. In some mechanisms, such as artificial neural networks, the components interact to such a degree that system behaviour can only be understood in terms of the components' overall organization [13]. Moreover, mechanisms may interact (e.g. share a component). In such cases, the degree of intra- versus inter-system interactions will determine how successfully the system can be decomposed [18]. These intersections could offer opportunities for mechanistic integration. For example, the mechanism for protein synthesis was discovered by molecular biologists approaching the problem in a bottom-up manner and biochemists proceeding in a top-down manner until both groups met in the middle at RNA [19].

Though challenging, building multi-level explanations can be satisfying and increase confidence in findings across levels. For example, Hebbian learning (i.e. cells that fire together, wire together) requires a coincidence detector to gate learning, and this requirement is well matched to the operation of NDMA receptors [20]. This alignment both makes Hebbian learning appear viable and situates the function of NDMA receptors within an encompassing system.

## 2. What is gained and lost traversing levels

One key question is what is the relationship between different levels of analysis. Certainly, different levels of analysis provide different viewpoints on the same phenomena. However, different levels are not equivalent to one another. In particular, additional explanatory concepts arise at higher levels of analysis whereas lower levels of analysis contain more information.

The many-to-one mapping from higher level explanations to lower level explanations highlights that there is more information present at lower levels. For example, sorting as a computational account can be realized by countless different sorting algorithms at the algorithmic level. Knowing the algorithmic account provides more information (specificity) than is provided by the computational level account alone. Likewise, there are many ways in which a mental state could be realized by neural activity. For example, the same error term in a reinforcement learning model is consistent with many different firing patterns of striatal neurons. In the physical domain, the same temperature, which is an aggregate measure of kinetic energy, can arise from many configurations of particles. In economics, the same unemployment rate for a nation is consistent with many combinations of people in and out of work. The canonical example in computation is that a Turing machine can be implemented in many physical substrates, including the device from which you are reading this sentence, tinker toys [21] and chemical reactions [22]. In all these examples, the higher level account does not fix all the details of the lower level account, though it will usually constrain the space of solutions. For example, the space of algorithms that do not perform sorting is much larger than those that do.

Supervenience is a useful concept for understanding the relationship between levels of analysis [23]. Briefly, supervenience holds that a change in a higher level entity must involve a change in the lower level entity, but not vice versa. The classic example is the relationship between the mental and physical—there cannot be a change in mental state without some corresponding change in physical state. Likewise, the computational level goal in terms of desired input–output mapping cannot change while the underlying algorithm remains fixed. One quick note is that while supervenience is necessary for reduction, it may not be sufficient (for a stronger notion of ground, see [24]).

Although higher levels of analysis involve the loss of information, they can offer explanatory concepts that do not exist at lower levels of analysis. These higher level concepts are central to our understanding. For example, how could people make sense of the economy without higher level concepts such as unemployment, inflation, money supply, etc.? Likewise, how could neuroscience progress if we only referred to atoms or even neurons without any higher level conceptual organization?

In this light, the eliminative reductionist programme rarely seems to reach its destination. It is hard to imagine neuroscience without concepts like consolidation, receptive field, replay, learning, error, recognition, etc. These are all concepts that reside at a high level. It would seem as undesirable to eliminate these high-level concepts as it would to discuss computing applications in terms of nothing higher level than transistors, eschewing higher level concepts such as algorithms and programming languages. When our higher level concepts are proven incorrect, we seem more inclined to replace them with other higher level concepts rather than simply eliminate them. We might retain higher level concepts for reasons other than conceptual convenience as higher level concepts can be realized in multiple lower level forms (e.g. the Turing machine) such that fixing the lower level with no connection to a higher level concept could lead to an incomplete account of the domain.

One question is whether the information at one level can constrain theories at another level. Given that the relationship between levels is asymmetric in multiple ways (e.g. supervenience, loss of information at higher levels, additional concepts at higher levels), there are actually two cases to consider: (i) Does information at the higher level constrain the lower level? (2) Does information at the lower level constrain the higher level? The answers to these basic questions have ramifications for how neuroscientists evaluate explanations and can guide how formal models (often at the algorithmic level) are related to brain measures [25–29].

Considering the first case, the higher level can constrain, though not completely determine, the lower level given the one-to-many possible mappings from the higher level to the lower level. For example, knowing that an application performs sorting provides a constraint on the possible algorithms but does not specify the particular algorithm. The higher level information does provide a useful constraint, though. For example, by knowing sorting is being performed, one could evaluate a number of possible sorting algorithms and cleverly notice that their predicted runtimes differ in informative ways as a function of problem size. By conducting the appropriate experiments varying problem size and recording runtime (akin to response time in a psychology study), one could infer which algorithm from the set is most likely used. Here, knowing the application (at the higher level) constrained the search space at the lower level. Likewise, in model-based functional magnetic resonance imaging (fMRI), including the error term from a cognitive model in the general linear model (GLM) analysis may identify candidate neural activity related to error processing [30]. Of course, such a result would not prove those regions and not others are involved in error correction. Beyond the standard correlative concerns, there are a number of ways that information could be coded at lower levels that will not be discoverable by fMRI [31]. Nevertheless, this top-down approach provides valuable constraints in that it can rule out possibilities and focus the researcher on identifying which unknowns should be investigated.

Moving in the other direction, lower levels can constrain the higher levels, though the key concepts of interest have to already be present at the higher level to select among. The lower level, by definition, does not contain these concepts. For example, one can look at BOLD activity all day but there is nothing in this measure alone that will lead one to propose algorithmic concepts like prototype and exemplar models of categorization. However, assuming these higher level concepts are already established, one can ask whether BOLD activity is more consistent with one algorithmic account or another. I was involved in a model decoding paper that did just this and found that changes in brain state more closely tracked changes in the internal state of an exemplar model than a prototype model [32]. Behaviour alone did not favour one model over another for this task, so this was a case where lower level information was critical to selecting among competing higher level concepts.

For this kind of higher level model selection to work based on information at lower levels, there needs to be some continuity between levels [33]. The same can be said of joint modelling procedures that simultaneously relate measures from different levels to exploit shared variance and mutual constraints [25]. For example, the model selection procedure we formulated explicitly assumed continuity across levels. Our claim was that to the extent that an algorithmic level model is ‘real', its state should be reflected by brain state as measured by BOLD response. If the gulf between these two levels of analyses were greater, it's unlikely we would have been successful in bridging these levels.

A related criticism of computational accounts in cognitive science that are not readily computable (e.g. some rational Bayesian approaches) is that they cannot be easily put in tight correspondence with an actual algorithm [34]. This lack of correspondence is a barrier to multi-level explanation. One proposed solution is to use sampling approaches (reflecting cognitive constraints) to derive algorithmic versions of computational level theories. For example, in recent years, bounded rationality [35] has been repackaged as resource-rational analysis in which an intractable Bayesian rational account is approximated by an algorithmic level model through sampling [36]. The approximate model can show systematic deviations from the computational level theory that allow it to account for human decision biases, such as mimicking the anchoring-and-adjustment heuristic [37,38].

These discrepancies across levels bring into doubt whether resource-rational analysis is a multi-level explanatory framework. To make an analogy, is a sorting algorithm (figure 1) that does not properly sort (i.e. follow a computational level account of sorting) actually a sorting algorithm? More generally, it is not clear that an approximation can be said to be a lower level version of the intended computational level theory when the algorithm does not compute the function specified at the computational level [33,34]. Worryingly, the approximating algorithm does not strictly contain more information than its supposed higher level counterpart as information is lost in the approximation. Accordingly, supervenience does not hold. This discrepancy between levels can lead to theoretical inconsistencies in which other computational level accounts better match the output of the sampling algorithm than the computational level theory the sampling algorithm aims to approximate.

This lack of correspondence across levels in resource-rational analysis goes beyond a classic critique of algorithmic level models, namely that many possible algorithms could equally well compute the same computational level function [39]. Although true, that classic critique is odd given that lower level explanations should contain more information than their higher level counterparts, much like how a temperature could be realized by many configurations of particles. In the case of resource-rational models, the situation is worse in that the algorithmic level model may better match many alternative computational level accounts. The problem cannot be solved by adding resource constraints to the computational level to match the input–output behaviour of the algorithmic model because those constraints would involve assumptions about processing resources, which are not computational level considerations. It can also prove challenging to move in the opposite direction from an algorithmic level model to the corresponding computational level account, although it is possible in some cases [40].

One common-sense conclusion is that integration across near levels should be more prevalent and successful. One can see this sociologically as well. For example, in my experience, attendees at the Annual Meeting for the Society for Neuroscience are curious about adjacent fields, but not fields many steps away. This is probably sensible. To be more extreme, a breakthrough in string theory is unlikely to impact cognitive neuroscience.

## 3. The twin sirens of reduction and emergence

Discussion in neuroscience about what counts as a satisfying explanation are invariably tied to levels of analysis, and in particular to whether higher level phenomena can be reduced to a lower level account. Why study higher level concepts that in reality merely reflect some lower level mechanism? The flip side of this attitude is the suggestion that phenomena arise that are emergent and are impossible in principle to explain through lower level accounts. Consciousness (e.g. qualia, [41]) is a classic example of a supposedly irreducible entity. From the perspective of scientific practice, I will argue that both stances are misguided. In the rare cases where a perfect reduction is possible, the use or disuse of a level of analysis is largely a pragmatic issue, much like choosing to program in a higher level language (e.g. Python) versus assembly language. Likewise, as will be unpacked, scientists should avoid making strong metaphysical claims of emergence. Not only will such claims be contentious, they are also likely to be spurious because the conduct of science is chiefly guided by practical, epistemic concerns. For instance, practical limitations, such as the precision of measurement, characterization of initial conditions (e.g. butterfly effect), available computing resources, and the cleverness of researchers, will likely be the limiting factors on what can be reduced absent dubious ontological claims about emergence.

As touched on in the previous discussion of levels of mechanisms, neuroscience is a multi-level discipline whose purview ranges from ion channels to social behaviours [42]. One longstanding view is that a theory is reduced to another when the theory can be derived from the other using bridge laws [43]. Every entity in the reduced theory is converted into the other theory. Although this view itself is controversial, the practical prospects of full reduction in neuroscience seem fleeting. Most theories in neuroscience are not fully developed nor sufficiently formal to allow equivalencies to be established. Formal modelling work, such as response time modelling, is well placed to draw out such equivalencies within a level [44], but neuroscience as a whole seems insufficiently developed to seriously consider fully reducing behaviour to ion channels. Furthermore, as a matter of practicality, it's not clear what neuroscience would gain from this reductionist pursuit as the field would almost certainly still seek recourse in higher level concepts in practice, much like how classical mechanics persists despite being a special case or approximation of relativistic mechanics. Neuroscience is so broad and diverse and its boundaries shift to include topics such as neuroeconomics that it seems better suited to a level of mechanism approach to draw out connections and constraints across pursuits.

When scientists, even Nobel prize winners such as Robert Laughlin [45], state that some entity is emergent, it's often not clear what they are asserting. Are these claims that a phenomenon is sufficiently complex that for practical purposes it needs to be explained by appealing to higher level entities? If so, this is a practical matter, much like how psychologists study mental processes using higher level concepts while not denying that these mental processes supervene on the physical brain (i.e. there is nothing magical about thought). Or, are those claiming emergence (as their language often suggests) stating there is something special about what they study that is not reducible to lower level entities in principle?

The latter position is highly problematic in that it brings the physical basis of scientific explanation into question. It is beyond the scope of this paper to unpack the philosophical implications of strong or ontological emergence (see [46] for a review), but briefly the emergence of causally efficacious entities that cannot be reduced to their constituent parts can lead to downward causation and causal overdetermination [46]. For example, if mental states arise (i.e. supervene) on physical states and physical states cause one another (i.e. causal closure of the physical domain), then issues arise when emergent mental states themselves become causally potent. On many analyses, one ends up with what most scientists would regard as ‘magic' or epiphenomenal emergent properties, which would seem to run counter to the motivation for invoking emergence in the first place. This is an area of active debate within philosophy [47] that is very interesting, though neuroscientists are probably best served by not advancing strong forms of emergence as they will be on uncertain footing.

There are many phenomena in science that are labelled as emergent that in reality are reducible to their constituent parts, but happen to be difficult to reduce in practice. For example, swarm phenomena in which a bunch of locusts or birds form an emergent entity are readily modelled on a computer in which each entity (e.g. a locust) follows its own simple, local rules [48]. In this case, as in Conway's Game of Life, the emergent properties are reducible to lower level entities [49]. Related, chaotic phenomena are difficult to understand, but are deterministic simulations that are highly sensitive to initial conditions. Such phenomena may at times be irreducible in current practice, but are not irreducible in principle. In other words, these examples can be reconciled without invoking magic or bringing physicalism into doubt. What these and other examples do challenge is the ability of scientists to understand complex phenomena that involve many interacting elements, which is par for the course in neuroscience. This weak, epistemic emergence arises not from magic, but from our own ignorance, cognitive limitations and imperfect tools [50–52].

## 4. Beyond levels and biological plausibility

Neuroscientists often invoke *biological plausibility* to support certain accounts over others. A search of ‘biologically plausible' on Google scholar returns 103 000 hits. Biological plausibility is something the field strives toward and prizes. Unfortunately, it is not clear that the claim of biological plausibility has content or is coherent, particularly in the context of multi-level theorizing. Are higher level descriptions never biologically plausible and if so, why? Alternatively, can one go too low toward physics and no longer be biologically plausible? These questions are intended to highlight how poorly conceived and empty neuroscience's notion of biological plausibility is and the confusion that results. For example, neural network models are both praised and criticized in different quarters for being and not being biologically plausible.

Asserting biological plausibility would seem to presuppose the answer to the research question. If neuroscientists could easily judge what is biologically plausible, then we would not need to do further research. In practice, the claims are often empty. For example, early connectionist models were characterized as biologically plausible whereas production systems, like Adaptive Control of Thought-Rational (ACT-R) [53], were characterized as implausible. As far as I can tell, these early connectionist models, which in the vast majority of cases did not make contact with actual brain data, were biologically plausible because they had a bunch of units with connections that did stuff and the brain also had a bunch of stuff that did stuff. Meanwhile, ACT-R has actually been used in model-based fMRI analyses to help understand brain activity that unfolds over seconds during complex tasks, such as mental arithmetic [54].

**Figure 2.**
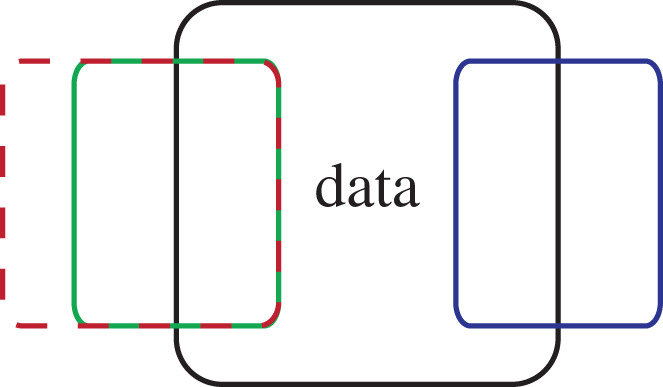
Models should be preferred to the extent that they predict and only predict the true data patterns. A model selection procedure should prefer the green model over the dashed-red model because both models capture the same findings but the dashed-red model is consistent with more events that do not occur. The red model is more flexible, related to the common (and not always correct) criticism that a model with enough parameters can fit anything. The interesting case is the green versus blue model. Both models are equally complex (i.e. flexible) but account for different aspects of the data. Claims of biological plausibility can amount to advocating for the green or blue model from no firm basis. (Online version in colour.)

To be charitable, when neuroscientists claim biological plausibility it is possible they are quietly entertaining some empirical finding that is consistent with their preferred model rather than a vague unsubstantiated intuition. If so, to make the claim substantive, the relevant data should be specified so that model selection procedures can determine the best account. In model selection, the model that is most likely given the data is preferred, which in practice means choosing the model that balances data fitting and complexity (e.g. number of parameters) or alternatively has the best cross-validated performance. In figure 2, notice that the green and dashed-red models both fit the same portion of the observed data, while also predicting outcomes outside what is observed. The red model is more flexible (i.e. complex) in what it can predict and is therefore less likely given the data than the green model, which should be preferred over the red model.

Notice that the green and the blue models are equally complex and equally fit the data, albeit different aspects of the data. Which of these models is more biologically plausible? Imagine the green model is a deep learning model of object recognition (trained through backpropagation) that best fits recordings from the ventral visual stream. Imagine the blue model is a model of object recognition, but one that uses a ‘biologically plausible' learning rule inspired by apical dendrites of pyramidal neurons [55–57]. In summary, the green model is not concerned with apical dendrites of pyramidal neurons, whereas the blue model does not capture activity along the ventral stream very well. Being generous, the meaning of biologically plausible from common usage appears to be ‘fitting the data one values'. The model selection addresses this impasse because it requires researchers to specify the datasets of interest, sidestepping vacuous, underspecified or misleading claims of biological plausibility.

In the previous example, the blue and green models would not be competitors if the empirical findings chosen to evaluate each model were non-overlapping. In the case shown in figure 2 in which all the data in the black box are deemed relevant, the blue and green models do equally well overall by capturing different aspects of the data. Further empirical investigation and model testing would be necessary to prefer one model over the other.

One might misconstrue biological plausibility as some top-down, theoretical judgement and mistakenly cast model selection as a bottom-up, data-driven approach. This dichotomy does not hold because model selection involves making important theory-guided choices, such as choosing the relevant datasets to explain, the relevant findings or constraints to follow (e.g. the spiking rate of artificial neurons should not eclipse the maximum rate observed in actual neurons) and the competing models to evaluate. Moreover, any claim of biological plausibility that had substance would itself need to be rooted in some finding, known constraint, or dataset, which if properly stated and evaluated would closely conform to model selection.

The term biological plausibility should be dropped and instead researchers should clearly state the relevant datasets they intend to address with their theory or model. Adopting this model selection orientation should also foster appreciation that accounts exist at different levels. For example, it would be very strange to attack a high-level cognitive model for lacking ion channels, as would it be strange to attack the Hodgkin–Huxley model for not accounting for human categorization behaviour under dual-task conditions. Once the explanandum (e.g. a relevant study) is clear and competing models are evaluated, claims of biological plausibility do no additional work. The model that fares best in model selection is the most ‘biologically plausible' for the phenomena of interest.

One speculation is that levels in scientific enquiry, which are not engineered as in the computer case (figure 1), arise from communities interested in certain types of datasets. In effect, the community is linked by repeated model selection on overlapping datasets such that eventually a theoretical language arises that is suited to describing the relevant phenomena, an idea not far off from Kolmogorov complexity. Adoption of a shared theoretical language would further cement social bonds and define the community.

## 5. Measurements are not levels

One question is whether neural measurements that reflect the activity of many cells, such as BOLD, are at a different level of analysis from finer grain measures, such as single-unit recordings. The brief answer is that measures themselves are not levels of analysis, but that different measures can be appropriate for evaluating mechanisms at different levels of analysis. For example, the temperature is an aggregate measure of the kinetic energy of particles in some region. Thus, a thermometer will suffice for evaluating a theory that only makes recourse to temperature, such as Boyle's Law. By contrast, a thermometer would not suffice for evaluating the Maxwell–Boltzmann distribution for the speed of particles for which finer grained measurements are needed. In other cases, the scale of the measurement itself determines its applicability. For example, measuring moderate speeds is fine for evaluating Newtonian mechanics whereas measuring incredible speeds is needed for Relativistic mechanics.

The relationship between BOLD and the firing rate of cells within a region does not appear to be a matter of simple aggregation as it is with particle speeds and temperature. If it were, then BOLD could be used as a thermometer for neural activity without further consideration. Although there is a relationship between BOLD and neural activity [58–60] that has enabled advances in cognitive neuroscience, the interpretation of BOLD response is not always straightforward [61]. For example, BOLD response is affected by the local vascular anatomy [62,63], differs according to age [64], and certain regions are susceptible to imaging artefacts. The fact that BOLD does not simply reflect aggregate neural activity (either synaptic or spiking) complicates its usage.

On the positive side, BOLD's divergence from simple aggregation presents some opportunities. Perhaps rather than just reflecting grey matter activity, BOLD may also reflect white matter [65–67] and astrocyte [68–71] activity as well. If so, BOLD response may track some general notion of energy consumption that could be useful for evaluating theories. Because BOLD is such an important measure for evaluating higher level neuro-computational accounts, research that can explain what BOLD is measuring should help clarify exactly what higher level accounts applied to BOLD are telling us about the brain [61].

Even though measures are not levels of analysis, the same chauvinism seems to reign in which researchers' preferred measure is proclaimed to be fundamental. Of course, there is not a fundamental measure, just as there is not a fundamental level of analysis. Certainly, finer grain measures, both in terms of spatial and temporal resolution, would be desirable. However, even if we had the magic machine that recorded every aspect of every cell at every millisecond, we would still need higher level accounts to make sense of this data deluge. In this scenario, higher level accounts would likely aggregate over the fine-grain measures, as we already do to an extent when preprocessing BOLD data.

## 6. Discussion

A grasp of levels of analysis is key to scientific progress. For better or worse, the day-to-day conduct of science is shaped by scientists' understanding of levels. Notions of mechanism, emergence and reduction, even what one considers a satisfying explanation, are all tied to levels. How scientists construe the relationship between their work and others' is tied to levels. A poor understanding of levels can lead to incoherent claims of biological plausibility and unsubstantiated beliefs that what one studies is somehow fundamental. These misconceptions can slow scientific progress by obscuring where the true fault lines and uncertainty lie.

There is not a single accepted hierarchy of levels, nor needs there be a fixed number of levels. Indeed, in the levels of mechanism approach, a component of a mechanism can itself be further decomposed. For example, the heart is part of the circulatory system but can be decomposed into its own parts that support its function. Notice that the concept of a heart can still be useful and treated as real even though it can be further decomposed. In general, scientists would be better served by considering how explanations relate to one another and evaluating whether explanations are useful rather than engaging in metaphysical debates about the ontological status of entities at levels above particle physics. This is especially true in neuroscience, where every entity is above this base level.

In this contribution, I have tried to make clear the relationship between different levels of analysis. Different levels are not equivalent. There are a number of asymmetries between lower and higher levels. By definition, lower levels contain more information whereas higher levels introduce additional explanatory concepts that can be useful, even in cases where they can be reduced. The particular concepts and terms may change over time as theories change, but neuroscience will always appeal to higher level concepts, such as consolidation, receptive field, replay, learning, error and recognition. Should all neuroscientists stop appealing to higher level concepts, they will no longer be neuroscientists but will instead be chemists, physicists, etc., and neuroscience will cease to be an active discipline. Scientists should resist the temptation to label every level above their preferred level as superfluous and every level below as involving uninteresting details.

For issues involving reduction and emergence, scientists are advised to focus on practical, epistemic concerns. Although many neuroscientists have a reductionist bent [17], the majority of theories in neuroscience are not sufficiently developed nor formalized to allow for reduction. At the same time, neuroscientists are surprisingly tolerant of claims of emergence, which can bring physicalism into question. Many phenomena that are labelled as emergent can actually be simulated on a computer through local interactions of the lower level entities, such as in swarm behaviour. For phenomena that we can't explain through lower level interactions in practice, scientists should be open to the possibility that epistemic factors, such as limits in measurement, computation or their own ability, are the limiting factors to understanding. It's not clear what the scientific rationale is for wading into the choppy philosophical waters of strong emergence.

To build a level of mechanism understanding, scientists need to determine how various explanations relate, such as whether explanations are competing, unrelated, or at different levels. Unfortunately, claims of biological plausibility do not achieve these ends and are incoherent under a level of mechanism view. A charitable interpretation is that claimants of biological plausibility have some dataset in mind that their model addresses that some other model does not. By instead specifying the relevant data, model selection could be performed to determine the best model without recourse to vacuous claims of biological plausibility, which both presupposes the form of the solution and are largely in the eye of the beholder. Model selection requires specifying the relevant datasets, which bears a resemblance to specifying the level of analysis, though model selection can be both narrower (e.g. just one dataset) and broader (e.g. datasets crossing levels), as well as less ambiguous. Claims of biological plausibility offer no value beyond what can be gained through model selection.

Measures, such as BOLD, are themselves not levels of analysis but are often confused as such. For example, one common assertion is that there is a behavioural level of analysis. Although Marr's computational level can be concerned with behaviour, it is in the context of a task specification (e.g. the input–output mapping, which could be the stimulus-response mapping for a task). Behaviour, BOLD response and single-unit recordings are all dependent measures that can be used to evaluate theories. Like levels, there is not some fundamental measure and understanding how measures relate to one another can be fruitful. One path to progress in neuroscience is exploiting the mutual constraints across different levels of analysis and measures.

## Data accessibility

This article does not contain any additional data.
